# Regional variation in distal femur subchondral bone mineral density: An in vitro human cadaveric model

**DOI:** 10.1016/j.jor.2025.08.003

**Published:** 2025-08-09

**Authors:** Kyle W. Zittel, Kyle P. Zielinski, Madison C. Thompson, William F. Postma, Ryan S. Murray, Bryan W. Cunningham

**Affiliations:** a Department of Orthopaedic Surgery, MedStar Georgetown University Hospital, 3800 Reservoir Rd NW, Washington, DC, 20007, USA; b Georgetown University School of Medicine, 3900 Reservoir Rd NW, Washington, DC, 20007, USA; c Musculoskeletal Research and Innovation Center, Department of Orthopaedic Surgery, MedStar Union Memorial Hospital, 201 E University Pkwy, Baltimore, MD, 21218, USA

**Keywords:** Bone mineral density, Osteochondral allograft transplantation (OCA), Osteochondral autograft transplantation (OAT), Osteochondral defect

## Abstract

**Background:**

Osteochondral defects of the knee are a frequent cause of pain that often require surgical intervention. Restorative procedures such as osteochondral autograft transplantation (OAT) and osteochondral allograft transplantation (OCA) aim to replace cartilage lesions with healthy autologous tissue or cadaveric tissue from the distal femur, respectively. Osteochondral graft donor sites are selected to optimize donor-recipient site congruity by assessing factors such as surface topography, cartilage thickness, and contact pressures. However, few studies have evaluated subchondral bone mineral density (BMD) when selecting an osteochondral graft donor site.

**Purpose:**

To examine bone morphology and characterize variation in subchondral BMD of the human distal femur.

**Study design:**

Descriptive Laboratory Study.

**Methods:**

18 human cadaveric distal femurs were utilized in the current study. All soft tissue and ligaments of the distal femur were removed. Dual-energy X-ray absorptiometry (DEXA) scans were performed on each femur to determine BMD of five regions of interest (ROI). ROIs included the entire distal femur, medial condyle, lateral condyle, posterior condyles, and trochlea. Subsequently, 90 osteochondral bone plugs (10 mm diameter, 10 mm depth) were harvested collectively from the medial and lateral trochlea of included specimens. DEXA scans of each osteochondral graft were then performed. Statistical analysis was performed via Welch Two-Sample *t*-test. Significance was defined as p < 0.05.

**Results:**

All specimens were included for analysis. There were no significant differences in the dimensional characteristics or BMDs of corresponding ROIs across distal femurs. There was no significant difference in the BMD of the medial condyle compared to the lateral condyle, and the posterior condyle compared to the trochlea. Also, there was no significant difference in BMD of grafts extracted from the medial and lateral trochlea (all p > 0.05).

**Conclusion:**

No significant difference was found in BMD between the medial and lateral condyles, trochlea and posterior condyles, or osteochondral grafts harvested from the medial and lateral trochlear ridges of the distal femur.

**Clinical relevance:**

Similar BMD between common osteochondral donor and recipient sites may provide surgeons with added confidence that fewer adjustments must be made to optimize congruity with respect to bone density.

## Highlights

• **Quantitative Density Assessment:** DEXA scans used to assess BMD across five regions of interest of the distal femur and osteochondral plugs.
• **Similar Subchondral Bone Density Across Regions:** No significant differences in subchondral bone mineral density (BMD) among regions of interest.
• **Clinical** **Implication**: Fewer adjustments must be made for bone density congruity during osteochondral transplantation procedures.

What is known about the subject: Osteochondral graft donor sites are selected by assessing factors such as surface topography, cartilage thickness, and contact pressures to optimize donor-recipient site congruity.

What this study adds to existing knowledge: No studies have evaluated subchondral bone density in the distal femur in the context of osteochondral graft transplantation. This study evaluates BMD of the distal femur which can be used to better inform osteochondral donor-recipient site selection.

## 1 Introduction

Full-thickness articular cartilage lesions are an important cause of pain that are difficult to manage in the young, active patient population.^1^, ^2^, ^3^ The gold standard treatment for symptomatic focal chondral defects is surgery.^3^ Among surgical treatment options, restorative procedures such as osteochondral autograft transplantation (OAT) and osteochondral allograft transplantation (OCA) aim to replace cartilage lesions with healthy autologous osteochondral tissue or fresh frozen cadaveric donor tissue, respectively.^4^ Osteochondral autograft transplantation avoids burdens associated with cadaveric tissue allocation and decreases the risk of an adverse immunologic response or disease transmission.^5^ Autologous osteochondral plugs are harvested from relatively non-weight bearing surfaces in the knee, specifically the trochlear ridge or femoral condyles, to minimize donor-site morbidity.^3^ Congruity between the osteochondral graft and recipient site is necessary to reduce contact pressure, point loading, and restore native joint articulation.^6^^,^^7^ As such, osteochondral graft donor sites are selected by assessing factors such as surface topography, cartilage thickness, and contact pressures to optimize donor-recipient site congruity.^8^, ^9^, ^10^, ^11^ Some animal studies suggest that subchondral bone mineral density (BMD) should also be considered for osteochondral graft donor site selection.^12^^,^^13^ However, few studies have evaluated subchondral BMD when selecting an osteochondral graft donor site in humans.^14^ Therefore, the purpose of this study is to examine bone morphology and characterize variation in subchondral BMD of the human distal femur.

## 2 Methods

### 2.1 Specimen preparation

Eighteen human cadaveric distal femurs were utilized in the current study. All soft tissue and ligaments of the distal femur were removed. Each specimen underwent preliminary radiography in anterior-posterior (AP), lateral, and notch views to confirm the absence of pre-existing osseous pathology and surgical history. No specimens were excluded. Cadaveric specimens were stored at −20 °C and were thawed to room temperature prior to testing.

### 2.2 Data collection

**Fig. 1:**
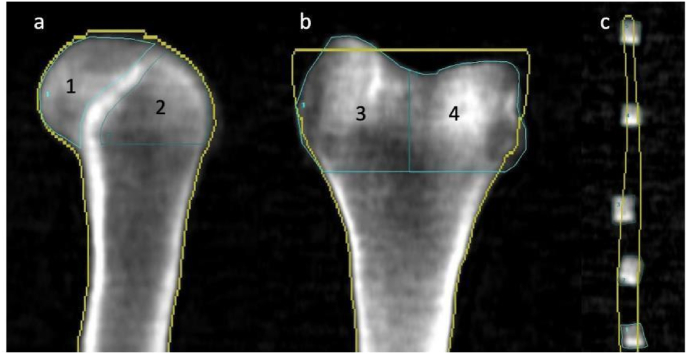
Example of DEXA scan technique with ROIs of the distal femur and osteochondral graft plugs. a. Lateral view used to measure posterior condyles (1) and trochlea (2) b. AP view used to measure medial condyle (3), lateral condyle (4), and entire distal femur (3 + 4). c. Harvested plugs.

The medial and lateral femoral condyles were measured and characterized in terms of their dimensions along the cephalocaudal, mediolateral, and anteroposterior (AP) axes. Dual-energy X-ray absorptiometry (DEXA) scans in the AP and lateral views were obtained for each specimen using a Lunar Prodigy Scanner 8743 (GE Medical Systems, Waukesha, WI). Bone mineral density (g/cm^2^) was obtained for five ROIs: the entire distal femur, medial condyle, lateral condyle, posterior condyle, and trochlea. The AP view was used to determine the BMD of the entire distal femur, medial condyle, and lateral condyle. The lateral view was used to measure the BMD of the posterior condyles and trochlea. The posterior condyle ROI was defined as superimposed medial and lateral posterior condyles on the lateral DEXA scan and Blumensaat line used to delineate the trochlea and posterior condyles (Fig. 1).^15^

**Fig. 2:**
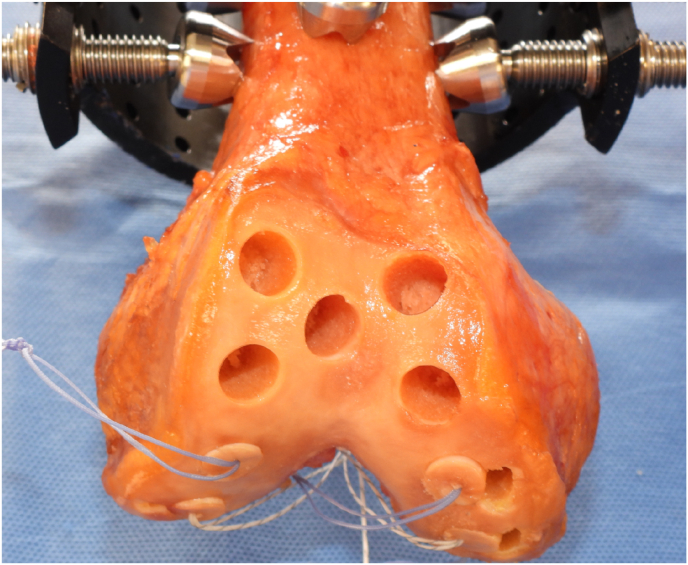
Example of distal femur with 5 osteochondral graft harvest sites from the proximal trochlea.

Following DEXA scans of each intact distal femur, 90 osteochondral grafts were obtained from the distal femur specimens, collectively. A total of 5 osteochondral grafts were harvested from a pre-templated location on the medial and lateral trochlea of each specimen (Fig. 2).

**Fig. 3:**
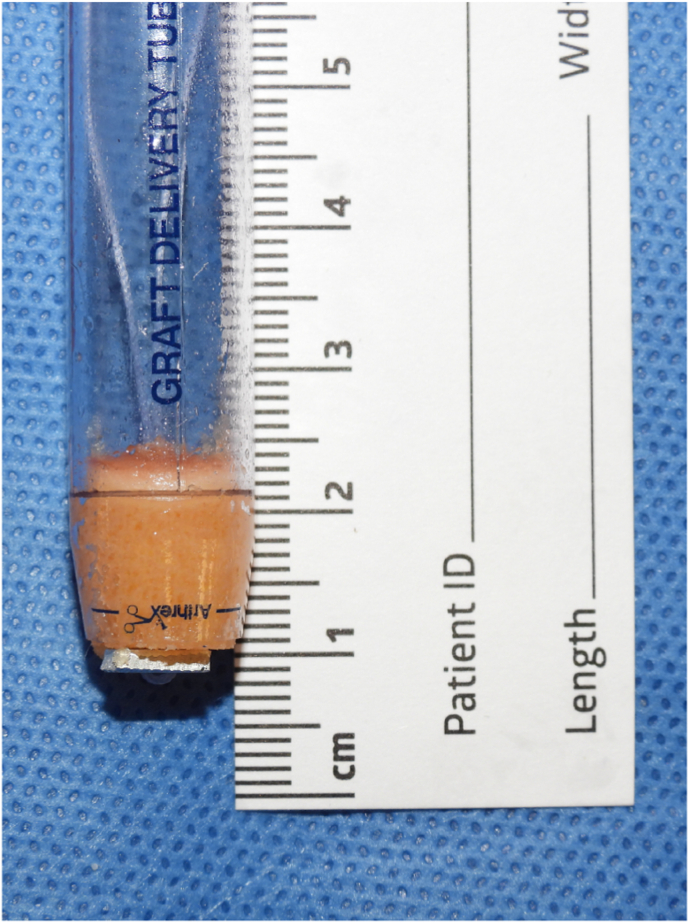
Example of osteochondral graft harvested with Autograft OATS 2.0 System (Arthrex, Inc., Naples, FL).

Each individual osteochondral graft, measuring 10 mm diameter and 10 mm depth (Fig. 3), was extracted in standard surgical fashion using a single-use osteochondral autograft transplant harvester.

Osteochondral grafts were labeled according to the ROI and location from which they were harvested. DEXA scans and the BMD of each individual osteochondral graft was then measured and recorded.

### 2.3 Statistical analysis

Comparisons between anatomic data were made utilizing One-Way Analysis of Variance (ANOVA) and post-hoc analysis via Tukey's method to test for group comparisons. Significance was set at p < 0.05.

## 3 Results

Ten male and eight female specimens were included for analysis. The mean age was 78.6 (range: 43–98) years. Average dimensions (Table 1) and BMDs (Table 2) of the distal femurs are provided below. There were no significant differences in the dimensional characteristics or BMDs of corresponding ROIs across femurs, confirming consistency of the sample (p > 0.05 for all comparisons).

**Table 1:** Dimensional characteristics of distal femurs; cm, centimeters; SD, standard deviation; A-P, anteroposterior; C-C, cephalocaudal.

Parameter	Measurement (cm), mean ± SD)
Medial condyle	
Length	7.44 ± 0.75
Width	2.56 ± 0.34
Lateral condyle	
Length	7.03 ± 0.53
Width	2.81 ± 0.30
Trochlea	
Length	3.89 ± 0.76
Width	4.69 ± 0.49
Trans-epicondylar	8.25 ± 0.87
A-P	6.44 ± 0.38
C-C	4.14 ± 0.92

**Table 2:** Mean BMD for regions of interest of the distal femur; BMD, bone mineral density; g, grams; cm, centimeters; SD, standard deviation.

Region	BMD (g/cm^2), mean ± SD
Entire distal femur	0.82 ± 0.22
Medial condyle	0.74 ± 0.23
Lateral condyle	0.91 ± 0.23
Trochlea	1.00 ± 0.32
Posterior condyles	1.19 ± 0.35

**Fig. 4:**
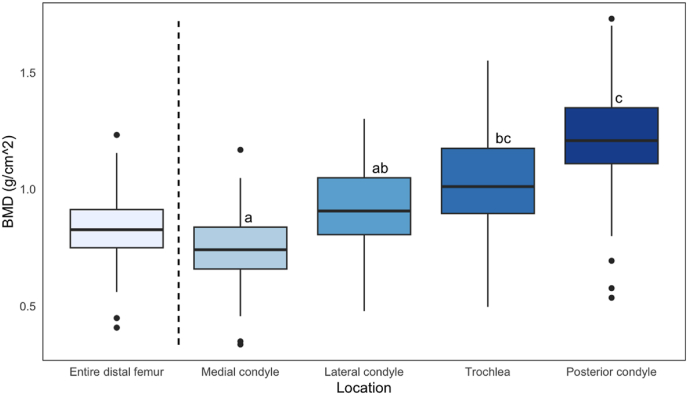
Comparison of BMD for ROIs of the distal femur. Matching letters indicate no significant difference (p > 0.05) between regional BMDs as determined by post-hoc analysis with Tukey's method. Differing letters indicate a significant difference in BMD (p < 0.05); BMD, bone mineral density.

The BMD of the medial condyle compared to that of the lateral condyle demonstrated no significant difference (p = 0.61) (Fig. 4).

Similarly, no difference was found between the posterior condyle and trochlea (p = 0.26). Lastly, no difference in BMD was found between plugs extracted from the medial trochlea and the lateral trochlea (p = 0.06) (Table 3).

**Table 3:** Bone mineral density of osteochondral plugs harvested from medial and lateral trochlea; BMD, bone mineral density; g, grams; cm, centimeters; SD, standard deviation.

Location	Plug BMD (g/cm^2), mean ± SD
Medial trochlea	0.20 ± 0.06
Lateral trochlea	0.23 ± 0.06

## 4 Discussion

The structural requirements of skeletal reconstruction should be considered when using autologous or allogeneic graft tissue. The purpose of this study was to examine bone morphology and characterize variation in subchondral BMD of the human distal femur to understand and optimize congruence between osteochondral graft donor and recipient sites. The analysis performed in this cadaveric model demonstrated no significant difference in BMD of the medial and lateral condyles, posterior condyles and trochlea, or amongst individual grafts harvested from medial and lateral trochlea. These findings may be useful to surgeons considering the subchondral bone quality of potential osteochondral graft harvest or recipient sites for OCA and OAT.

Osteochondral grafts are frequently harvested from the lateral femoral condyle (LFC), medial femoral condyle (MFC), intercondylar fossa, or trochlea.^16^, ^17^, ^18^, ^19^, ^20^ When allograft tissue from a fresh frozen cadaveric distal femur is utilized for transplantation, donor tissue is often allocated from MFC or LFC in isolation secondary to cost and availability, whereas autologous osteochondral grafts are most often harvested from the patient's ipsilateral knee at the lateral trochlea. However, symptomatic cartilage lesions and subsequent recipient sites in the knee are found most frequently on the MFC.^18^ Some studies have demonstrated that the use of osteochondral grafts harvested from the LFC could be used to treat MFC chondral defects due to similar surface characteristics.^17^^,^^18^ A study of osteochondral graft donor and recipient site topography by Urita et al. found no significant differences in articular cartilage surface mismatch and subchondral surface mismatch between LFC grafts and MFC defects.^18^ Notably, few studies have explored the importance of bone mineral density in osteochondral donor-recipient congruence. The findings of the current study demonstrate no significant difference in BMD between cadaveric distal femur LFCs and MFCs which suggests that the donor-recipient congruence in BMD would be similar at each respective site.

We further characterize subchondral BMD of the entire distal femur with specific ROIs which may be useful when evaluating suitability for alternative osteochondral donor and recipient sites throughout the knee. This information can also be useful for osteochondral grafting procedures at other articular surfaces throughout the human body, with potential for application to the already existing indications for OCA and OATs procedures for cartilage lesions in the elbow and ankle joints.

Subchondral bone density has been measured and used by some authors to identify areas of decreased load bearing.^14^ A study by Nishida et al. found subchondral BMD to be the lowest in the proximal lateral trochlea in a cohort of baseball players which may indicate areas of reduced load bearing that are more suitable for osteochondral autograft harvest.^14^ Using the rationale described by Nishida et al. on trochlea BMD and donor site morbidity, our analysis revealed no difference in subchondral BMD throughout this region which suggests that loading, or donor site morbidity after osteochondral autograft harvest, would be similar regardless if taken from the medial or lateral trochlear ridges.^14^ Our findings may instead indicate that the articular contact pressures and load bearing across a native patellofemoral joint have limited correlation with the subchondral BMD in that region. Still, it is important to recognize that donor site morbidity is a multifactorial and patient-specific issue. Factors such as patellofemoral tracking, trochlear dysplasia, level and location of medial and lateral patellar facet engagement within the trochlear groove during both knee flexion and extension should all be considered to determine appropriate graft harvest sites that minimize postoperative donor site symptoms.

Adjustments for bone quality should be made when harvesting osteochondral allograft or autograft plugs, particularly in areas of nonhomogeneous bone morphology, pathology, and density. Bone healing after transplantation occurs via creeping substitution, a slow process of bone remodeling by osteoblastic bone formation and osteoclastic resorption. In a study of fresh osteochondral allograft transplantation, Williams et al. reported that at six months postoperative, trabecular incorporation allograft bone was complete in three (17 %), partial in eleven (61 %), and poor in four (22 %) of 18 grafts based on MRI evaluation.^21^ Based on these findings, it was suggested that minimizing the amount of transplanted bone may reduce the healing time.^21^

Unlike subchondral bone, the hyaline cartilage component of a graft is mature and does not undergo further healing. It has been shown that through synovial joint fluid and diffusion, donor chondrocytes can survive for years. In a study by Jamali et al., authors detected surviving female donor allograft chondrocytes in a male host after 29 years.^22^

Radin and Rose have cited increased subchondral bone density and bone stiffness as factors that contribute to hyaline cartilage degeneration in the early stages of osteoarthritis.^23^ Using a live animal model, Lane et al. also concluded that increased stiffness seen after integration of osteochondral grafts with greater bone density may accelerate cartilage degeneration at the transplant surface.^24^

In contrast, inferior bone quality or low BMD may negatively affect the stability of press-fit graft fixation which can result in graft micromotion, graft subsidence, subchondral cystic cavitation.^25^ Graft subsidence is common, occurring in up to 30 % of patients.^26^ The lasting effect on the cartilage surface congruency and resultant level of clinical significance is likely under reported in existing literature and generally unknown. Recent short term follow up studies utilizing OCA demonstrate graft survival rates ranging from 79 % to 100 % between 2 and 4 years.^21^^,^^27^^,^^28^ Kock et al. found OCA survivorship to be 82 % at 10 years, 74 % at 15 years, and 66 % at 20 years.^29^ In a study of 60 patients who received distal femoral osteochondral allografts, the clinical results and survivorship were considered to be very encouraging at an average of 10 years follow up.^30^ Authors cite 12 of 60 patients (20 %) with graft failures at long term final follow up. Interestingly in 9 of the 12 reported failures, the grafts united to host bone with no fragmentation or resorption, and instead failed at greater than 5 years as the result of late osteoarthritic degeneration of the joint and complete loss of cartilage from the grafts.^30^ Late graft failure may be a result of the aforementioned findings published by Lane and Radin where relative graft stiffness and possibly increased BMD may accelerate chondral wear after osseointegration.^23^^,^^24^

Our findings demonstrate a similar distribution in BMD between medial and lateral femoral condyles, and medial and lateral trochlear ridges, which may provide surgeons with added confidence that fewer adjustments must be made when selecting between these regions for osteochondral graft harvest. To our knowledge, this is the first and largest human cadaveric study to analyze the BMD of the distal femur. Ultimately, the effects of BMD on donor-recipient site congruity have yet to be studied in a human clinical setting. Future research studies may benefit from inclusion of BMD analysis as it relates to clinical outcome measures, graft failure versus successful incorporation, and further study to develop techniques to ensure successful outcomes for patients undergoing OCA or OAT for articular cartilage restoration.

The following limitations should be considered for this study. First, the measurement of BMD using DEXA did not allow for absolute measurement of volumetric BMD in superimposed ROIs, as measured with qCT.^31^ Second, while this in vitro study is the largest cadaveric sample size analyzing BMD of both that of the distal femur and individual osteochondral grafts, a larger sample size would decrease the risk of type I statistical error. Finally, the current study results may not be directly generalizable to clinical practice without additional in vivo data. Therefore, further studies are warranted to validate our findings.

## 5 Conclusion

No significant difference was found in BMD between the medial and lateral condyles, trochlea and posterior condyles, or osteochondral grafts harvested from the medial and lateral trochlear ridges of the distal femur. Our findings suggest that BMD is similar between the MFC and LFC, and medial and lateral trochlear ridges in this in-vitro human cadaveric knee model.

## CRediT authorship contribution statement

**Kyle W. Zittel:** Conceptualization, Data curation, Formal analysis, Funding acquisition, Investigation, Methodology, Project administration, Supervision, Writing – review & editing. **Kyle P. Zielinski:** Data curation, Project administration, Validation, Visualization, Writing – original draft, Writing – review & editing. **Madison C. Thompson:** Data curation, Formal analysis, Writing – review & editing. **William F. Postma:** Conceptualization, Funding acquisition, Project administration, Supervision, Writing – review & editing. **Ryan S. Murray:** Conceptualization, Funding acquisition, Project administration, Supervision, Writing – review & editing. **Bryan W. Cunningham:** Conceptualization, Data curation, Formal analysis, Funding acquisition, Investigation, Methodology, Project administration, Resources, Software, Validation, Visualization, Writing – review & editing.

## Guardian/patient's consent

Not Relevant.

## Funding/sponsorship statement

The authors received no financial support for the research, authorship, and/or publication of this article.
